# Effects of a Standardized Community Health Worker Intervention on Health Care Utilization Within an Integrated Delivery System

**DOI:** 10.1007/s11606-025-09495-6

**Published:** 2025-04-11

**Authors:** Aditi Vasan, Molly Knowles, Shaun Flerchinger, Thomas K Tandy, David Mosen, Jessica Soltesz, Olivia Paradis, Nicole Friedman, Judith A. Long, Briar L. Ertz-Berger, Shreya Kangovi

**Affiliations:** 1 Division of General Pediatrics, University of Pennsylvania Perelman School of Medicine, Philadelphia, PA USA; 2 Penn Center for Community Health Workers, University of Pennsylvania Health System, Philadelphia, PA USA; 3 Division of General Internal Medicine, University of Pennsylvania Perelman School of Medicine, Philadelphia, PA USA; 4 Kaiser Permanente Northwest Population Health, Portland, OR USA; 5 Kaiser Permanente Center for Health Research, Portland, OR USA; 6 Center for Health Equity Research and Promotion, Corporal Michael J. Crescenz VA Medical Center, Philadelphia, PA USA; 7 IMPaCT Care, Philadelphia, PA USA; 8 PolicyLab, Children’s Hospital of Philadelphia, Philadelphia, PA USA

**Keywords:** Community health workers, Social determinants of health, Health care utilization

## Abstract

**Background:**

Community health worker (CHW) interventions can improve health outcomes and reduce acute care utilization. Few prior studies have examined the association of CHW interventions with health care utilization among patients within an integrated health system.

**Objective:**

To evaluate the effects of Individualized Management for Patient Centered Targets (IMPaCT), a standardized CHW intervention originally developed within a single health system in Philadelphia, PA, on acute care utilization and primary care engagement among low-income patients at two clinics within an integrated health system in Portland, Oregon.

**Design:**

Prospective randomized analysis using adjusted difference-in-differences regression.

**Participants:**

In total, 1230 adults living in low-income zip codes were randomized using a 2:1 allocation sequence to receive either IMPaCT (*n* = 820) or usual care (*n* = 410).

**Interventions:**

IMPaCT is a standardized intervention in which CHWs use an in-depth interview to understand patients’ strengths, social needs, and health-related goals and then collaboratively develop tailored action plans. Over 3 months, CHWs communicated with patients at least once weekly to provide coaching, social support, and navigation tailored to their goals. Due to the COVID- 19 pandemic, the intervention was predominantly delivered remotely.

**Main Measures:**

Primary outcome measures were hospital and emergency department (ED) utilization, both measured per 1000 members per month, and proportion of patients with 1+ primary care visits. Implementation fidelity and maintenance were also assessed.

**Key Results:**

Compared to usual care, patients who received IMPaCT had a relative reduction in total hospital days at 6 months (− 172.3 days per 1000 members per month, 95% CI − 320.05 to − 24.53, *p*= 0.022), and a greater proportion attended 1+ primary care visits (85.7% vs. 79.5%, *p*= 0.006). There were no differences in ED utilization.

**Conclusions:**

A standardized CHW intervention delivered remotely within an integrated health system during the COVID- 19 pandemic was associated with decreased hospital utilization and improved primary care engagement.

## INTRODUCTION

Community health workers (CHWs) are frontline public health workers who share cultural identities, demographic characteristics, and lived experiences with the patients they serve and are therefore well positioned to improve health outcomes and promote health equity for low-income and minoritized patients.^1,2^ CHWs can play a key role in building trust between patients and health systems, improving access to care, and facilitating connections to social service programs and community resources.^3–5^ CHW interventions have been found to improve patients’ experience of care, reduce acute care utilization, and promote more equitable health outcomes.^6–12^

Many prior studies of CHW interventions have focused on implementation within a single health system, making it difficult to determine how these interventions can be effectively scaled across settings.^13–15^ Individualized Management for Patient Centered Targets (IMPaCT) is a standardized, theory- and evidence-based CHW intervention that has been shown to improve quality of care, reduce hospitalizations, and generate a positive return on investment in three prior randomized controlled trials.^6–8,16^ These initial studies evaluated implementation of IMPaCT at academic primary care clinics, Veteran’s Affairs health system clinics, and federally qualified health centers, all in Philadelphia, PA.

In this program evaluation, we adapted and implemented IMPaCT within Kaiser Permanente Northwest (KPNW), an integrated health care delivery system located in Oregon and Southwest Washington. We aimed to reduce acute care utilization and improve primary care engagement among low-income and minoritized patients at risk of disparities, while implementing the intervention with high fidelity. To our knowledge, this is the first study examining the effects of a standardized CHW intervention on health care utilization within an integrated care delivery system. Because integrated health plans serve as both provider and payer, they are uniquely incentivized to increase engagement with preventive care, improve chronic disease control, and reduce acute care utilization. In addition, due to the global COVID-19 pandemic, we had to rapidly adapt the IMPaCT intervention to be delivered remotely, rather than through in-person home and clinic visits, making this the first study to evaluate implementation of IMPaCT when delivered predominantly remotely. To assess utilization, we examined hospitalizations and emergency department utilization in the 6, 9, and 12 months after enrollment, and primary care utilization, measured by the proportion of patients with 1+ primary care visits in the 6 months after enrollment. To assess implementation, we evaluated intervention fidelity and maintenance.

## METHODS

### Evaluation Design, Setting, and Participants

This program evaluation used a randomized, prospective design with continuous recruitment and was conducted at KPNW, a non-profit integrated health care system that provides comprehensive health care to more than 630,000 members, owns and operates two hospitals, contracts with six other hospitals, and maintains 52 clinics. The KPNW team selected two primary care clinics serving a predominantly low-income population in Portland, OR, as study sites. The KPNW Population Health team (including authors J.S., N.F., and B.L.E.) approved and supervised hiring and integration of CHWs into clinic teams. There were a total of nine CHWs, one manager, and one program director involved in implementation. CHW and supervisor salaries were paid by KPNW operational funds. The KPNW Institutional Review Board reviewed this program evaluation and deemed it to be non-human subjects research.

Eligible participants were age 18 or older, empaneled to one of the study primary care clinics, lived in a low-income zip code with 30% or more of the population having incomes < 250% of the federal poverty level, and had a Johns Hopkins Adjusted Clinical Group (ACG) score ≥ 0.15 for white patients and ≥ 0.10 for patients of color. The ACG score parameter was set lower for patients of color because one of KPNW’s goals in implementing IMPaCT was to promote health equity. To achieve KPNW’s health equity goals, the evaluation coordinator preferentially outreached Black, Latine, Asian American/Pacific Islander, and American Indian patients, continuing until all eligible and interested patients of color had been enrolled.

### Randomization

Patients meeting eligibility criteria were offered the opportunity to enroll in the intervention via text message or phone call from the evaluation coordinator. Patients who chose to enroll were randomized using an allocation sequence in a 2:1 ratio to receive the CHW intervention or usual care. Participants were enrolled from July 2020 to July 2021, and health care utilization data reflect the time period from July 2019 (12 months before the first patient enrolled) through July 2022 (12 months after the last patient enrolled).

### Intervention

Patients randomized to usual care continued receiving typical care with no changes. For patients randomized to the intervention arm, CHWs implemented the IMPaCT intervention. To adapt IMPaCT to the KPNW system (Table 1), our team used a standardized implementation approach including constituent-engaged planning and adaptation involving local health system leaders, health care providers, and CHWs; development of tailored workflow manuals and tools for CHWs and supervisors; and structured guidance on CHW hiring, training, and supervision.^17^ As KPNW’s adaptation of IMPaCT began during the global COVID- 19 pandemic, this process also required adaptation to the rapidly changing contexts in which health care was being delivered. For example, in March 2020, the IMPaCT team worked with KPNW to develop workflows for remote intervention delivery via telephone calls and text messaging rather than in-person home visits.

**Table 1:** IMPaCT Model and Summary of Key Intervention Adaptations

	Original IMPaCT studies	KPNW intervention adaptations
*Initial patient assessment*	CHWs meet patients in-person during a hospital or clinic visit and complete an in-depth semi-structured interview, focused on understanding their strengths, goals, and unmet social needs	CHWs completed their initial in-depth semi-structured assessment interviews over the phone, rather than in-person, due to the COVID- 19 pandemic
*Goal-setting and action planning*	CHWs ask patients what they believe they need to improve their health and well-being and use participants’ individualized goals as the basis for tailored action plans	Actions plans were tailored both based on patients’ overall goals for improving their health and well-being, and based on new or emerging health-related needs specific to the COVID- 19 pandemic
*Personalized support from CHWs*	CHWs provide coaching, social support, advocacy, and navigation to support patients in achieving their health-related goals. CHWs attempt to communicate with all participants at least once weekly throughout the intervention period, attend clinic visits with patients when possible, and visit patients if they are admitted to the hospital during the intervention	CHWs provided coaching, social support, advocacy, and navigation through once weekly phone or text message contacts with patients, rather than in-person visits. Some emergent needs were addressed through home visits with social distancing protocols in place, for example, socially distanced delivery of food boxes, masks, or sanitizer
*Connection with long-term supports*	At the conclusion of the intervention, CHWs attempt to connect all patients with resources and long-term social supports that can continue to meet their needs	CHWs connected all patients with long-term resources and supports when possible, including virtual resources and support groups
*CHW hiring and training*	All CHWs were newly hired as members of the IMPaCT intervention team, according to detailed hiring protocols focused on prioritizing key traits, including empathy, non-judgement, listening skills, and reliability. All CHWs received standardized training	Some existing KPNW patient navigators completed standardized IMPaCT CHW training and then transitioned to a CHW role. Other CHWs were newly hired and trained according to IMPaCT protocols
*CHW safety protocols*	CHWs and supervisors followed detailed safety protocols aimed at both ensuring CHWs’ safety when interacting with patients and ensuring that any concerns about patients’ safety raised through their interactions with CHWs were addressed appropriately	Additional safety protocols were put into place to ensure CHW safety in light of the COVID- 19 pandemic, such as protocols for socially distanced home visits when needed
*Intervention duration and CHW caseload*	In one study, community health workers followed patients for 6 months, and each CHW had a caseload of approximately 55 patients annually, or 25 to 30 patients at a time. In another study, CHWs followed patients for 2–4 weeks, and each CHW had a caseload of about 90 patients annually	Community health workers followed each patient for 3 months, and each CHW had a caseload of approximately 20 to 28 patients at a time, with a yearly goal of 110 patients per CHW
*CHW support and supervision*	CHWs receive regular supervision and support from master’s level social workers, primarily through in-person interactions. CHWs also regularly collaborate and share insights with one another through in-person interactions	CHW supervision and support took place through phone and video calls, rather than in-person interactions. Collaboration and peer support relationships between CHWs also occurred predominantly through phone and video calls

CHWs began with an in-depth semi-structured interview focused on understanding patients’ strengths, goals, and social needs (e.g., food insecurity, housing instability, drug and alcohol use, family stress). As a part of this conversation, CHWs asked patients, “What do you think you need to improve your health and well-being?” Participants’ individualized goals for improving their health became the basis for tailored action plans. Over the subsequent 3 months, CHWs provided coaching, social support, advocacy, and navigation to support participants in achieving their health goals. CHWs attempted to communicate with all participants at least once weekly throughout the intervention period. Each CHW served patients at a single clinic, and all patients in the intervention arm interacted with a CHW at least once.

As this intervention was implemented during the COVID- 19 pandemic, most communication between CHWs and enrolled participants occurred through calls and text message. Some emergent needs were addressed through home visits, with protocols in place to ensure the safety of both CHWs and participants. For example, CHWs provided socially distanced delivery of food boxes to a subset of participants who were experiencing acute food insecurity. At the conclusion of the intervention period, CHWs attempted to connect all patients with resources and long-term social supports that could help continue to meet their needs.

### Health Care Utilization Outcomes

The primary outcome measures were hospitalization, measured as total hospital days per 1000 members per month, and emergency department (ED) utilization, measured as total ED visits per 1000 members per month, in the 6 months following randomization. Hospitalization and ED utilization were also assessed at 9 months and 12 months post-randomization. As a secondary outcome, we assessed proportion of patients who attended one or more primary care appointments at 6 months following randomization.

### Statistical Analysis

We used descriptive statistics to compare baseline characteristics for patients who were randomized to receive the CHW intervention and those who received usual care, including chi-squared tests for categorical variables and Kruskal-Wallis tests for continuous variables. In our analysis of health care utilization, we used a difference-in-differences approach to account for secular trends in health care use during the evaluation period. We felt that accounting for these secular trends was particularly important because the evaluation period spanned the first year and a half of the COVID- 19 pandemic, which was associated with changing patterns of illness, health care access, and health care utilization for all patients.^18,19^ We used linear regression with an intervention by time period interaction term to estimate the relative difference in hospitalization and ED utilization rates over time for patients who received the IMPaCT intervention, as compared to those who were randomized to usual care. We used multivariable regression models that adjusted for patient-level characteristics that might influence health care utilization, including age, sex, race, ethnicity, primary language, and Johns Hopkins Resource Utilization Band score. We recognize that race and ethnicity are social constructs and adjusted for these factors as a proxy for shared experiences of racism and discrimination among those in minoritized groups, which we hypothesized might impact their health care utilization. Our primary analysis focused on outcomes 6 months after enrollment, and as additional analyses, we examined outcomes at 9 months and 12 months after enrollment. For our secondary outcome, we used a chi-squared test to compare the proportion of patients who had 1+ primary care visits completed by 6 months post-enrollment.

### Implementation Outcomes

To monitor fidelity, the IMPaCT team conducted virtual direct observations of CHWs, supervisors, and program directors at each site 12 months after initial implementation. Each observation was scored on a 7-point scale (0–6), and these scores were averaged to create a global fidelity score. To assess maintenance, we assessed whether the intervention was maintained 3 years after initiation and whether it was expanded to serve additional populations.

## RESULTS

**Figure 1:**
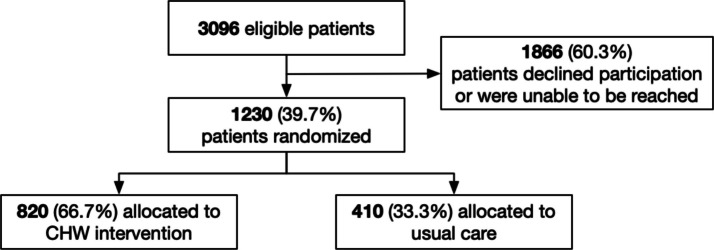
Consort diagram. A total of 3096 patients were identified as eligible for the IMPaCT intervention. Of these patients, 1866 declined participation or were unable to be reached. The remaining 1230 patients were randomized with 2:1 allocation, such that 820 received the CHW intervention and 410 received usual care.

A total of 3096 patients were identified as eligible for the intervention. Of these patients, 1866 were not enrolled, either because they were unable to be reached by phone or because they declined participation. The evaluation cohort included the remaining 1230 participants, with 820 randomized to receive the IMPaCT intervention and 410 randomized to receive usual care (Fig. 1).

Baseline demographic characteristics are presented in Table 2. Across both the intervention and usual care groups, patients were predominantly female and white, and spoke English as their primary language, reflecting the demographic characteristics of patients served by KPNW in this region. Most patients had a Johns Hopkins Resource Utilization Band score of 4 or 5, indicating high or very high morbidity related to chronic conditions. As compared to the usual care group, patients randomized to the intervention group were younger (mean age 61.6 vs. 64.0, *p* = 0.017), less likely to speak English as their primary language (87.6% vs. 92.9%, *p* = 0.004), and had higher rates of ED utilization at 6 and 12 months prior to randomization (185.5 vs. 151.3 visits per 1000 at 6 months, *p*= 0.022; 165.6 vs. 146.0 visits per 1000 at 12 months prior, *p* = 0.020). Patients randomized to receive the IMPaCT intervention were also disproportionately from minoritized racial and ethnic groups (16.5% vs. 9.5% Black, *p* = 0.003; 10.6% vs. 7.6% Hispanic/Latino, *p* = 0.088). There were no statistically significant differences in other baseline characteristics.

**Table 2:** Baseline Demographic Characteristics of Intervention Patients and Randomized Control Patients

Population characteristic	Intervention *N*= 820	Usual care *N*= 410	*p*-value
*Age (mean ± SD)*	61.6 (17.1)	64.0 (16.3)	*p* = 0.017
*Sex (n (%))* Female (vs. male)	523 (63.8%)	262 (63.9%)	*p* = 0.967
*Language (n, (%))* English (vs. non-English)	718 (87.6%)	381 (92.9%)	*p* = 0.004
*Race (n, (%))*			*p* = 0.003
American Indian or Alaska Native	16 (2.0%)	6 (1.5%)	
Asian	43 (5.2%)	15 (3.7%)	
Black or African American	135 (16.5%)	39 (9.5%)	
Native Hawaiian/Other Pacific Islander	16 (2.0%)	6 (1.5%)	
White	505 (61.6%)	300 (73.2%)	
Other	1 (0.1%)	0 (0.0%)	
Unknown or declined to state	104 (12.7%)	44 (10.7%)	
*Ethnicity (n, (%))*			*p* = 0.088
Hispanic/Latino	87 (10.6%)	31 (7.6%)	
Non-Hispanic/Latino	733 (89.4%)	379 (92.4%)	
*Johns Hopkins Resource Utilization Band N (%)*			
3	71 (8.7%)	53 (12.9%)	*p*= 0.065
4	299 (36.5%)	140 (34.1%)	
5	447 (54.5%)	217 (52.9%)	
*Prior ED utilization (6 months prior)*			
Adj. ED rate per 1000 members per month	185.5 (289.0)	151.3 (307.4)	*p*= 0.022
*Prior hospital utilization (6 months prior)*			
Adj. IP Days per 1000 members per month	279.8 (937.3)	200.6 (704.9)	*p*= 0.237
*Prior ED utilization — 12 months prior*			
Adj. ED rate per 1000 members per month	165.6 (224.0)	146.0 (276.0)	*p*= 0.020
*Prior hospital utilization — 12 months prior*			
Adj. IP days rate per 1000 members per month	229.2 (701.4)	205.5 (589.0)	*p*= 0.959

Adjusted difference-in-differences estimates for relative change in health care utilization are presented in Table 3. As compared to control patients, patients who received the IMPaCT intervention had a relative reduction in hospital days at 6-months post-enrollment (− 172.3 days per 1000 members per month, 95% CI − 320.05 to − 24.53, *p*= 0.022). More specifically, the total number of hospital days per 1000 members per month decreased by 78.8 days in the CHW intervention group and increased by 92.7 days in the control group over this time period. This corresponds to 1032 fewer hospital days per 1000 KPNW members over a 6-month period. At 9- and 12-months post-enrollment, the relative reduction in total hospital days for patients who received the intervention was maintained but was no longer statistically significant (9 months: − 124.69 days per 1000 members per month, 95% CI − 265.93 to 16.54, *p* = 0.084; 12 months: − 109.45 days per 1000 members per month, 95% CI − 248.80 to 29.90, *p* = 0.124). These reductions correspond to 1122 fewer hospital days per 1000 members over a 9-month period and 1313 fewer hospital days per 1000 members over a 12-month period, respectively. There were no statistically significant differences in ED utilization at 6, 9, or 12 months.

**Table 3:** Adjusted Differences-in-Differences Estimates of Relative Change in Health Care Utilization

	Diff-in-Diff estimate	95% CI	*p*-value
Total population, 6 months (*N*= 1230)			
-Adjusted ED visits per 1000 members per month	− 15.38	(− 67.09, 26.34)	*p*= 0.560
-Adjusted hospital days per 1000 members per month	− 172.3	(− 320.05, − 24.53)	*p*= 0.022
Total population, 9 months (*N*= 1230)			
-Adjusted ED visits per 1000 members per month	− 16.60	(− 63.32, 30.13)	*p*= 0.486
-Adjusted hospital days per 1000 members per month	− 124.69	(− 265.93, 16.54)	*p*= 0.084
Total population, 12 months (*N*= 1230)			
-Adjusted ED visits per 1000 members per month	− 6.21	(− 51.28, 38.85)	*p*= 0.787
-Adjusted hospital days per 1000 members per month	− 109.45	(− 248.80, 29.90)	*p*= 0.124

Compared with patients who received usual care (Table 4), a greater proportion of patients who received the IMPaCT intervention had attended one or more primary care visits (85.7% vs. 79.5%, *p* = 0.006) at 6 months post-enrollment.

**Table 4:** Rates of Primary Care Visit Completion

	Intervention *N*= 820	Usual care *N*= 410	*p*-value
% of patients with 1+ primary care visits	703 (85.7%)	326 (79.5%)	*p*= 0.006

We found that IMPaCT was implemented with high fidelity in direct observations with 15 program staff members across best practice domains such as hiring, patient-centered CHW workflows, supervision, safety, and clinical integration. Across all domains, KPNW achieved a mean global fidelity score of 4.6 out of 6.0. Three years after implementation, KPNW had sustained their CHW program at the two original clinics and expanded to serve patients at a third primary care clinic and on the general medicine floors at two hospitals.

## DISCUSSION

In this program evaluation, we examined the effect of an adapted, remotely delivered version of the standardized IMPaCT CHW intervention on health care utilization within an integrated health care delivery system, finding a reduction in hospital days per 1000 members per month and an increase in primary care engagement at 6 months post enrollment, as compared to patients receiving usual care. These findings are in line with previous studies of the IMPaCT intervention, which found that this structured, theory- and evidence-based approach to addressing social determinants of health and supporting patients in meeting their health-related goals was associated with significant reductions in acute care utilization.^6–8,20^

These findings, along with implementation outcomes showing high fidelity and successful maintenance and expansion, suggest that by using a standardized approach to intervention tailoring and adaptation, we were able to implement a version of the intervention that maintained fidelity to the IMPaCT model, met the unique needs of the KPNW system and their patient population during the COVID- 19 pandemic, and achieved health care utilization outcomes similar to those of the original IMPaCT RCTs in Philadelphia.

Although patients who received the IMPaCT intervention had a significant reduction in hospital days at 6 months post-randomization, this difference was no longer statistically significant at 9 or 12 months. This could suggest that the effects of the intervention decreased in the 6 to 9 months after patients stopped working with their CHW, or that we were not adequately powered to detect smaller differences in long-term utilization. These findings may also have been impacted by secular trends in health care utilization during the COVID- 19 pandemic. One prior study found reductions in hospitalizations for both ambulatory-care sensitive and non-ambulatory sensitive conditions during the pandemic.^21^ Overall reductions in acute care utilization may have made it more challenging to detect statistically significant between-group differences at 9 and 12 months. It is also possible that the 3-month duration of the intervention in this evaluation was not sufficient to generate longer-term improvements in health care utilization; previous studies of the IMPaCT intervention showed persistent decreases in health care utilization when patients received 6 months of CHW support.^8,20^

We saw no significant relative changes in ED visits per 1000 members per month. This suggests that patients in the CHW intervention group were still seeking care in the ED at equal rates but may have had a lower likelihood of subsequent admission and shorter hospitalizations. This could indicate improved chronic disease control associated with the CHW intervention, in line with findings from previous studies of CHW programs.^20,22–26^ Decreased rates of hospital days despite equal rates of ED visits could also suggest that ED and inpatient teams who were aware of the intervention may have been more comfortable with discharging intervention patients home, knowing that they were receiving or had received longitudinal CHW support focused on addressing their social needs.

Our findings on primary care engagement similarly suggest that the CHW intervention may have reduced hospital utilization by increasing patients’ utilization of primary care. As CHWs are skilled in working across health systems and the surrounding communities and in building trust, they are uniquely positioned to increase engagement with primary care among low-income and minoritized patients. These results are in line with prior research which has shown that CHW programs can increase access to and utilization of primary care.^2,7^

Our analysis of implementation outcomes showed that KPNW was able to maintain high fidelity to the IMPaCT intervention structure, despite delivering the intervention remotely, and was able to sustain and scale the program 3 years after initial implementation. Barriers to implementation of evidence-based CHW programs in new health care settings may include difficulties with clinical integration and shifting health system finances and priorities, while facilitators include context-specific adaptation of intervention workflows.^17,27^ Our findings suggest that despite shifting models of care and health system priorities during the COVID- 19 pandemic, KPNW was able to implement and maintain the IMPaCT model in part through thoughtful adaptation of workflows that allowed CHWs to support participants remotely and address pandemic-specific needs.

Our findings suggest that as payers begin to reimburse for services provided by CHWs, they should consider reimbursement for services delivered remotely, via phone or text message, in addition to services delivered during hospital, clinic, or home visits, as remote interventions may be equally effective in reducing acute care utilization and improving primary care engagement.

These evaluation findings should be interpreted in light of several limitations. Although participants were prospectively randomized to receive either the intervention or usual care, there were notable baseline demographic and health care utilization differences between intervention and usual care patients. To minimize the effects of these differences, we used a difference-in-differences regression model that adjusted for baseline demographics and utilization. In addition, we did not measure the number of encounters each patient had with a CHW or assess for differences in utilization based on patients’ level of engagement with the intervention. We also did not assess acceptability of the intervention among enrolled patients. These are important areas for future study. This evaluation also has strengths, including use of an integrated health system dataset and use of difference-in-differences regression modeling to account for changing health care utilization patterns during the COVID- 19 pandemic.

## CONCLUSIONS

In this program evaluation, we find that implementation of IMPaCT, a standardized community health worker intervention, within an integrated care delivery system was associated with decreased hospital utilization and improved primary care visit attendance at 6 months. These results are despite the COVID- 19 pandemic and ensuing need to adapt the intervention to be delivered remotely. Our findings suggest that standardized CHW interventions may be able to achieve significant reductions in acute care utilization across a wide range of health systems and care settings, even when delivered remotely.

## Data Availability

The data that support the findings of this study are available from the corresponding author upon reasonable request.
